# The clinical elective course and its effects on medical students and graduates of Jordanian medical schools

**DOI:** 10.1186/s12909-022-03779-9

**Published:** 2022-10-11

**Authors:** Raed Al-Taher, Ruba Al-Ani, Abdullah Al-Ani, Mohammad Rashdan, Abdel rahman A. Al Manasra, Emad Aborajooh, Hamzeh Al-Balas, Hasan Al-Balas, Mahmoud Al-Balas, Maymoona Attiyat, Nuha Qasem

**Affiliations:** 1 Department of General Surgery, Division of Pediatric Surgery, School of Medicine, The University of Jordan, Amman, Jordan; 2 School of Medicine, The University of Jordan, Amman, Jordan; 3 Department of General Surgery, School of Medicine, The University of Jordan, Amman, Jordan; 4 Department of General Surgery, Faculty of Medicine, Jordan University of Science and Technology, Irbid, Jordan; 5 Department of General Surgery, Faculty of Medicine, Mutah University, Kerak, Jordan; 6 Department of General and Special Surgery, Faculty of Medicine, The Hashemite University, Zarqa, Jordan; 7 Clinical Medicine Department, Faculty of Medicine, Yarmouk University, Irbid, Jordan; 8 Department of General Surgery, Faculty of Medicine, Al-Balqa Applied University, Al-Salt, Jordan; 9 Department of Internal Medicine, Division of Family Medicine, Faculty of Medicine, The Hashemite University, Zarqa, Jordan

**Keywords:** Elective, International training, National training, Medical schools, Medical students, Undergraduates, Clinical training, Jordan

## Abstract

**Background:**

The elective course is part of the 6th-year medical school curriculum in Jordan. Students choose the specialty in which they wish to spend 8 weeks and choose their location even if it is outside their university’s affiliated hospitals. In this study, we try to understand student choices regarding the country of elective, chosen specialty, type of placement (observership/clerkship), and elective general value from participants' perspectives.

**Methods:**

This paper used a cross-sectional study. The survey was distributed through social media platforms (mainly Facebook and WhatsApp) targeting 6th-year medical students and doctors who graduated from one of the 5 Jordanian medical schools (the University of Jordan, Jordan University of Science and Technology, Mutah University, Yarmouk University, and Hashemite University).

**Results:**

The majority of participants had an international elective (69.6%), mainly in the USA, followed by the UK. Internal medicine was the primary field of interest for 14.8%, followed by general surgery. Of these, 241 (62.6%) actively participated in work at their chosen hospitals as they had a clerkship/hands-on experience. In contrast, 142 (36.9%) were observers. The majority indicated that the elective is worth the time, money, and effort. Moreover, they had adequate supervision throughout the course and could achieve their preset objectives.

**Conclusions:**

The elective course gives a unique experience to our students. General satisfaction is an indicator of the success of the course in actively exposing medical students to clinical practice.

## Introduction

The clinical elective is one part of the medical school curriculum, usually part of the last year of the medical school program. It is an elective course in terms of chosen specialty, country, and institution. Usually, the students would conduct their enrollment and study through different methods, including direct connections with consultants or applications through hospital websites [1].

The elective course has many valuable perspectives and is universally well received by students [2]. The clinical elective course aids most undergraduate students in gaining clear insight into their future postgraduate specialty [3, 4]. It allows medical students to engage in clinical practice and apply their knowledge. Thus, they improve their clinical experience and acquire more confidence to practice medicine [5].

Students in their clinical elective can be either passive observers or actively involved in multiple aspects of care and tasks, including clinical assessment, care management, or participation in invasive procedures [6].

A significant proportion of medical students worldwide participate in enriching, usually self-organized, clinical electives outside their home country, known as international clinical electives or global health electives. Students of international electives show a significant post-experience improvement from many perspectives, including personal, professional, and academic performance [7].

The international elective experience contributes favorably to student learning and career growth in several ways. First, it helps significantly in strengthening existing skills and learning new diagnostic skills. Second, attitudinal changes and ethical learning are significant, such as a greater appreciation of the importance of cross-cultural communication. Third, it influences the career choices of many medical students [8].

Clinical electives provide many benefits to medical students, but not all students get the same experience, and some find the elective course a waste of money, time, and effort. The clinical elective course in medical schools in Jordan takes approximately 8 weeks of the last academic year of medical training. However, its effects have not been studied or reviewed by medical school directors. Moreover, student feedback, struggles, and drawbacks have not been evaluated. Therefore, this study attempts to understand student preferences, including their preferred specialties, countries, and locations, and to assess the general satisfaction with elective course experience among Jordanian medical students and graduates. In this way, we can make a clearer account of the advantages and disadvantages of this course to make a baseline for future improvement.

Assessment of elective course effectiveness and outcomes were measured by analyzing questions directed to medical students about their experience from different perspectives [9].

Thousands of Jordanian medical students participate in "elective" courses each year, which are calculated as 8 credit hours in the scheduled degree plan; some schools limit the students to specific months during the sixth year, while others allow students to arrange their elective course at any time during the final year for a duration of 8 weeks. The elective is the best way for students to practice the discipline of their interest [1].

Furthermore, familiarizing the student with the diverse opportunities available and introducing them to the process of applying, enrolling, and preparing for attending the relevant course ameliorates and even optimizes student learning experiences [10].

The effectiveness of the elective course was measured across many universities in the USA, Canada, the Netherlands, Saudi Arabia, the UK, and other countries, which showed significant benefits. Additionally, in these studies, students had the opportunity to identify defects that might be reconsidered. Unfortunately, in Jordan, there are no studies that help us to understand and evaluate the elective course for medical students and how it can be improved to achieve the desired objectives [8, 11, 12, 3].

Our study aims mainly to assess the value of the clinical elective and identify factors that contribute to differences in student experiences so as to understand how students can take the greatest advantage of this opportunity.

## Methodology

### Study design

This was a cross-sectional study targeting 6th-year medical students in Jordan (class of 2020) and graduates of Jordanian medical schools (class of 2019 and before). The students and physicians who participated in this study were assessed using an online questionnaire survey in English that included quantitative questions and two qualitative questions. The link to the questionnaire was distributed on social media (Facebook, WhatsApp) to Jordanian medical groups using Google services. All health care workers and a class of 2020 medical students were invited to participate in this survey. Informed consent from each respondent was obtained before proceeding with the questionnaire.

### Instrumental development

The questionnaire consisted of informed consent as a first part, followed by six demographic-related questions (gender, age, university of graduation, year of graduation, current position, and whether the elective was international or national). Six questions addressed various aspects of the expected benefits of the elective course and solicited responses from students on a 5-point Likert scale ranging from 0—strongly agree to 4-strongly disagree. In addition, one question assessed the overall value of the elective, from 0—experience of no value to 5—experience of great value. Participants who took national electives were able to skip some questions directed to international electives automatically and vice versa. At the end of the questionnaire, there were two open-ended questions.

### Sample

The questionnaire had 385 responses. The questionnaire targeted graduates of Jordanian medical schools and 6^th^-year medical.

### Study subjects

#### Election criteria

- Participants > 20 years old
- Medical school graduates and 6^th^-year medical students of one of the six Jordanian medical schools (University of Jordan, Hashemite University, Yarmouk University, Mutah University, Jordanian University for Science and Technology).
- Class of 2020 and earlier.
- Participation from 21/05/2020 to 01/12/2020

#### Exclusion criteria

1. Participants < 20 years old.
2. 1^st−^, 2^nd−^, 3^rd−^, 4^th−^, and 5^th^-year medical students.
3. Graduates and medical students at non-Jordanian medical schools.
4. Class of 2021 and beyond
5. Participation after 01/12/2020

### Data analysis

Data were analyzed using SPSS version 23. Questionnaire items were reported as frequencies (percentages). Mean and standard deviation were used for age, general evaluation of elective from participants’ perspective out of 5, and level of satisfaction of each of the six items in Table 3 on a scale rating 0 as the minimum to 4 as the maximum (0-strongly disagree, 1-disagree, 2- neutral, 3- agree, and 4-strongly agree). The Kruskal‒Wallis test was used to find the correlation between the 6-item ratings and the 5-point scale for the general value of the elective in Table 2. A *P* value of less than 0.05 was considered significant. Crosstables were used to better understand the relation between placement location (national or international) and type of placement (observership or clerkship) with the general value of the elective. National elective participant responses were excluded while measuring the general value of the elective among international elective participants, as shown in Table 4, and vice versa using the “select cases; if” option in SPSS.

All responses to qualitative questions were added to a word document. In referring to the theme of responses we categorized the answers into 5 organizational subheadings (important advice that was repeated frequently, selection of location and specialty, on an individuals’ level, negative reviews about having an elective abroad). Then, we reviewed them in the results (qualitative analysis) and in the discussion.

### Ethical consideration

Institutional Review Board (IRB) approval was obtained at the University of Jordan in May 2020. Participation in this study was voluntary, and as mentioned clearly in the preface, "completion and submission of this form is considered approval to participate." All information gathered during the course of this study was confidential, and participants' anonymity was protected at all times. No questions were asked about the participant names or other personal information that could reveal the participants' identities. In addition, only the research team members were allowed to review the participants' responses.

## Results

### Demographics and elective setting

A total of 385 participants from different levels of experience completed the questionnaire. Table 1 shows that the mean age of the participants was 26.31 ± 4.072 years. The majority (122, 31.7%) were 6^th^-year medical students, followed by interns (118, 30.6%), residents, general practitioners, specialists, consultants, and fellows, all of whom graduated before 2021. Of these, 214 (55.6%) were male, and 171 (44.4%) were female. Overall, 35.6% of all participants studied at the University of Jordan. Most of the participants enrolled in an international elective (268, 69.6%) compared to 117 (30.4%) national electives.

**Table 1:** Table of demographics

Factor	Category	Frequency (%)	Mean ± SD
Age	23	47 (12.2%)	26.31 ± 4.072
	24	125 (32.5%)	
	25	86 (22.3%)	
	26	30 (7.8%)	
	27	15 (3.9%)	
	28	8 (2.1%)	
	29	11 (2.9%)	
	30	8 (2.1%)	
	> 31	55 (14.3%)	
Gender	Female	171 (44.4%)	
	Male	214 (55.6%)	
University of Graduation	University of Jordan	137 (35.6%)	
	Jordan University of Science and Technology	97 (25.2%)	
	Hashemite University	86 (22.3%)	
	Yarmouk University	40 (10.4%)	
	Mutah University	25 (6.5%)	
Year of Graduation	< 2015	64 (16.6%)	
	2015	10 (2.6%)	
	2016	4 (1.0%)	
	2017	7 (1.8%)	
	2018	27 (7.0%)	
	2019	123 (31.9%)	
	2020	150 (39.0%)	
Current Position	Last-Year Medical Student	122 (31.7%)	
	Intern	118 (30.6%)	
	General Practitioner	44 (11.4%)	
	Resident	49 (12.7%)	
	Specialist	29 (7.5%)	
	Fellow	8 (2.1%)	
	Consultant	15 (3.9%)	
National or International Elective	Inside Jordan	117 (30.4%)	
	Abroad	268 (69.6%)	

**Fig. 1:**
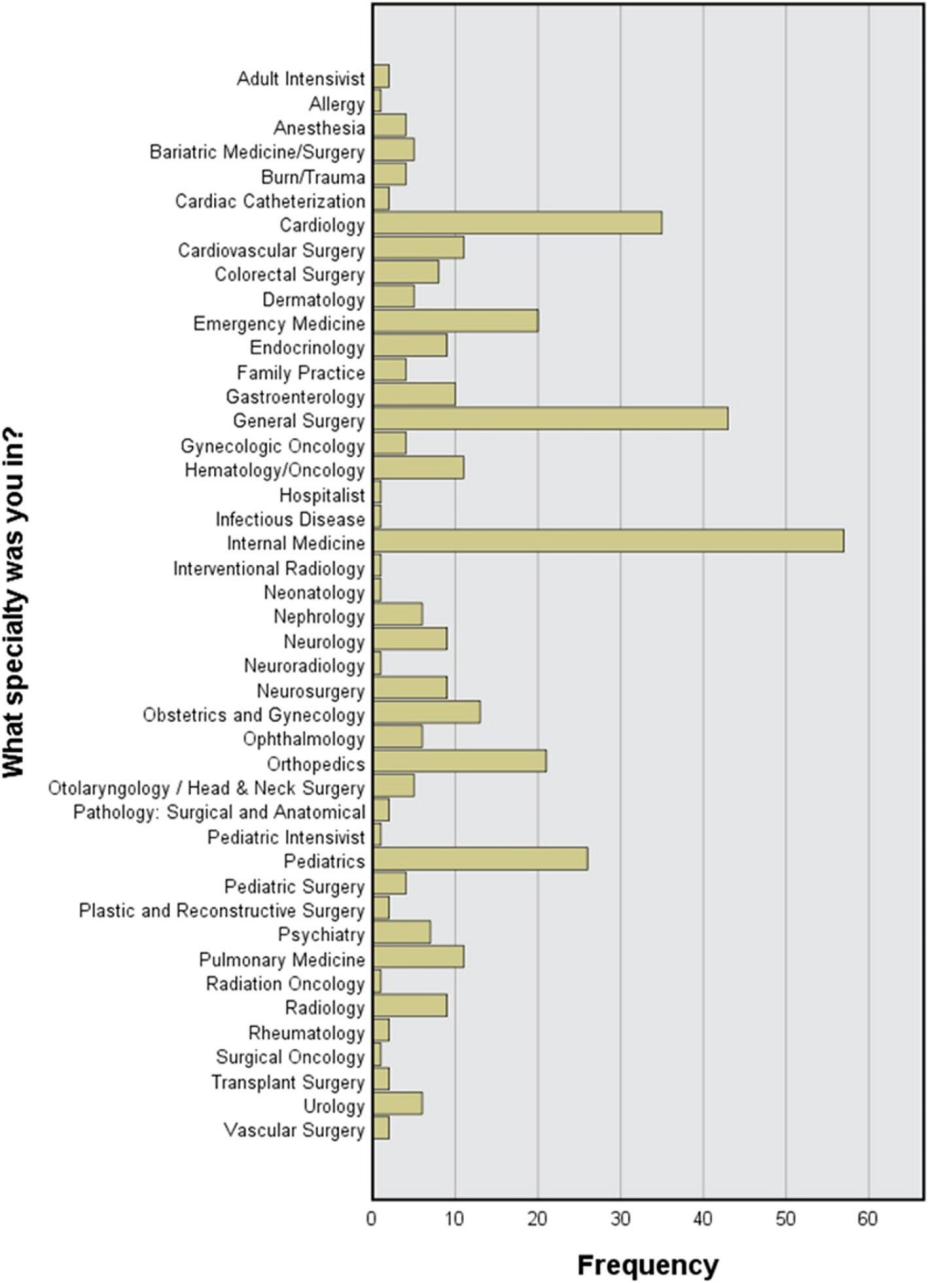
The Frequency of Chosen Specialties

Table 2 summarizes the elective choices, such as the type of placement, specialty chosen, the number of placements applied to for the elective course duration, the location of national electives or the country of international electives, and the method of obtaining the placement. A total of 241 (62.6%) were placed in clerkships, while 142 (36.9) were observers. Placements were in more than 40 specialties. The majority of them, 57 (14.8%), were in internal medicine, followed by general surgery with 43 (11.2%) (Fig. 1). The vast majority did more than one placement. For example, 134 (34.8%) did their first international placement in the United States of America, and 85 (22.1%) also had their second elective placement in the USA. The UK comes in second place with 31 (8.1%) participants, and Germany comes in third place with 23 (6%) participants. On the other hand, for those who had national electives, 43 (11.2%) performed their first placement at Ministry of Health hospitals, followed by 34 (8.8%) at the Royal Medical Services. Most participants used direct application to the concerned hospital in getting placement 192 (49.9%), while 106 (27.5%) guaranteed placement through connections (through friends or relatives*).*

**Table 2:** General ideas about the elective choices

	**Category**	**Frequency (%)**
**Type of placement**	Observership	142 (36.9%)
	Clerkship/Hands-on	241 (62.6%)
**Way of getting the placement**	Connections through friends or relatives	106 (27.5%)
	Exchange programs	32 (8.3%)
	Arrangement through home university	41 (10.6%)
	Arrangement with for-profit organization	11 (2.9%)
	Direct application to the concerned hospital	192 (49.9%)
	IFMSA	1 (0.3%)
	Chicago clerkship	1 (0.3%)
**Chosen Specialty**	Cardiology	35 (9.1%)
	Emergency Medicine	20 (5.2%)
	General Surgery	43 (11.2%)
	Internal Medicine	57 (14.8%)
	Orthopedics	21 (5.5%)
	Pediatrics	26 (6.8%)
	All other specialties	183 (47.4%)
**Number of placements -international elective**	1	108 (28.1%)
	2	144 (37.4%)
	> 3	16 (4.2%)
**Country of placement #1—international electives**	Germany	23 (6%)
	Kuwait	7 (1.8%)
	United Arab Emirates	14 (3.6%)
	United Kingdom	31 (8.1%)
	United States	134 (34.8%)
	All other countries	53 (13.9%)
**Country of Placement #2—international elective**	Germany	15 (3.9%)
	Turkey	4 (1%)
	United Kingdom	10 (2.6%)
	United States	85 (22.1%)
	All other countries	22 (6%)
**Number of placements completed—national elective**	1	51 (13.2%)
	2	53 (13.8%)
	> 3	13 (3.6%)
**Location of Placement #1—national electives**	Jordan University Hospital	13 (3.4%)
	King Abdullah University Hospital	14 (3.6%)
	King Hussein Cancer Center	1 (0.3%)
	Private Hospital	5 (1.3%)
	Public Health Center (Ministry of Health)	7 (1.8%)
	Public Hospital (Ministry of Health)	43 (11.2%)
	Royal Medical Services	34 (8.8%)
**Location of Placement #2—national elective**	Jordan University Hospital	7 (1.8%)
	King Abdullah University Hospital	2 (0.5%)
	Private Hospital	2 (0.5%)
	Public Health Center (Ministry of Health)	5 (1.3%)
	Public Hospital (Ministry of Health)	32 (8.3%)
	Royal Medical Services	17 (4.4%)

### The general value of the elective

**Fig. 2:**
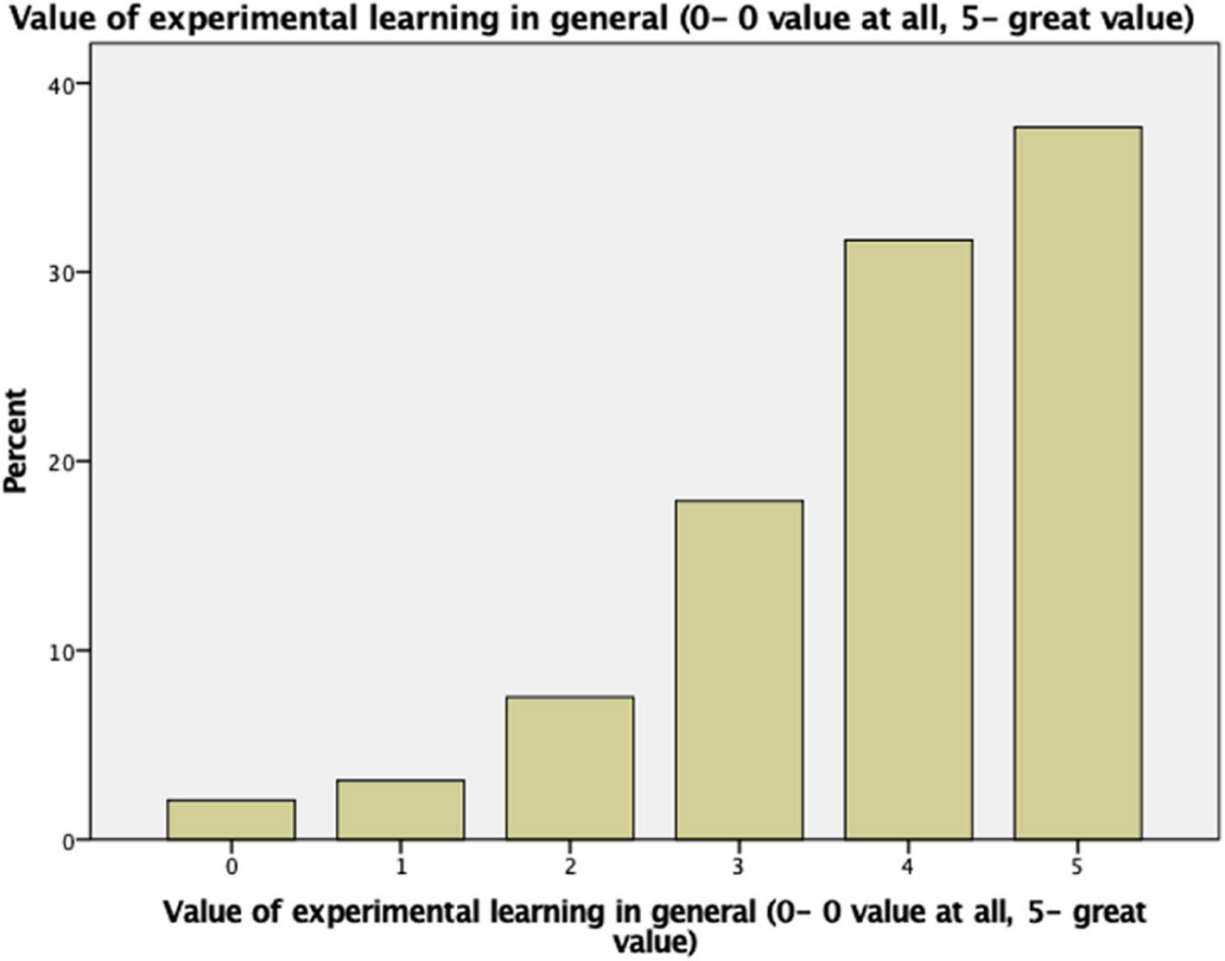
General value of the elective from the participants’ perspective on 5-point Likert scale (0- of no value, 5- of great value)

Table 3 illustrates the 6 items used to evaluate the value of the elective in general, which shows positive responses to the 6 questionnaire items. At the end of these items, participants were asked to evaluate the general value of the elective. A total of 69.2% evaluated their experience > / = 4 out of 5, with a mean (± S.D.) of 3.87 (± 1.207) Fig. 2. The correlation between these 6 items and the participants' evaluation of the general value of the elective is significantly correlated with a *P* value < 0.001 for all the items using the Kruskal‒Wallis test in Table 3.

**Table 3:** Participant Satisfaction with their Elective Experience

	Items	Strongly disagree, n (%)	Disagree, n (%)	/Neutral, n (%)	Agree, n (%)	Strongly Agree, n (%)	*P* value	Mean ± SD
**1**	I had adequate supervision during the clinical elective	13(3.4)	33(8.6)	64(16.6)	158(41)	117(30.4)	(< 0.001)	2.86 ± 1.05
**2**	I had adequate opportunities for hands-on clinical work (e.g., active involvement in the wards, clinics or operations, case presentation, etc.)	13(3.4)	42(10.9)	78(20.3)	121(31.4)	131(34)	(< 0.001)	2.82 ± 1.12
**3**	The elective provided me active learning through discussion/participation	11(2.9)	21(5.5)	63(16.4)	161(41.8)	129(33.5)	(< 0.001)	2.98 ± 0.988
**4**	The program was responsive to my needs (both academically and socially)	10(2.6)	36(9.4)	69(17.9)	161(41.8)	109(28.3)	(< 0.001)	2.84 ± 1.023
**5**	The elective was worth the time, effort and money	12(3.1)	35(9.1)	58(15.1)	136(35.3)	144(37.7)	(< 0.001)	2.95 ± 1.081
**6**	I was able to achieve the objectives I had set for myself	9(2.3)	36(9.4)	99(25.7)	152(39.5)	89(23.1)	(< 0.001)	2.72 ± 0.998

### General value of the elective and type of placement/location of placement

A comparison between the national and international general value of electives as rated by the participants is shown in Table 4. A total of 81.3% of international elective participants evaluated their experience > / = 4 out of 5 with a mean of 4.20 (± 0.985), compared to 47% of national elective participants with a mean of 3.15 (± 1.347). Regarding the type of placement (Table 5), 78% of those who did clerkships/had hands-on experience regarded it as valuable, as did 77% of observership participants.

**Table 4:** Correlation between the location of the elective and its general value

	General Value of the Elective (0–0 value at all, 5- great value)								
	0	1	2	3	4	5	Total	Mean (± SD)	*P* value
Inside Jordan	7 (6%)	7 (6%)	16 (13.7%)	38 (32.5%)	30 (25.6%)	19 (16.2%)	117 (100%)	3.15 (± 1.347)	< 0.001
Abroad	1 (0.4%)	5 (1.9%)	13 (4.9%)	31 (11.6%)	92 (34.3%)	126 (47%)	268 (100%)	4.20 (± 0.985)	
Total	8 (2.1%)	12 (3.1%)	29 (7.5%)	69 (17.9%)	122 (31.7%)	145 (37.7%)	385 (100%)	3.87 (± 1.207)

**Table 5:** Correlation between the type of elective and its general value

	General Value of the Elective (0–0 value at all, 5- great value)							
	0	1	2	3	4	5	Total	*P* value
Observership	5 (3.5%)	7 (4.9%)	16 (11.3%)	37 (26.1%)	46 (32.4%)	31 (21.8%)	142 (100%)	< 0.001
Clerkship/Hands-on	3 (1.2%)	5 (2.1%)	13 (5.4%)	32 (13.3%)	75 (31.1%)	113 (46.9%)	241 (100%)	
Total	8 (2.1%)	12 (3.1%)	29 (7.6%)	69 (18%)	121 (31.6%)	144 (37.6%)	383 (100%)	

### Qualitative analysis

One hundred fifty-seven participants responded to the first open question, while only 87 responded to the second. Some answers to these open-ended questions were repetitive of items already measured in the 5-point Likert score questions.

Responses to the first open-ended question, "What advice do you have for undergraduates who want to do an elective abroad?" were generally positive; almost all responses encouraged an elective experience abroad. However, 5 negative impressions about a clinical elective abroad were expressed among 157 responses, and the main complaint was that it was not worth the cost, followed by the language barrier. Most frequently repeated advice to undergraduates: take the risk and go abroad, set objectives beforehand, take the initiative, and plan it as early as you can. Some other advice on the location and the specialty choices included examples such as choose an elective in the country where you are planning to have your training in the future and in the specialty you prefer, try not to spend the whole duration of the elective in the same department or institution, apply to as many institutions as possible, accept rejections and do not fear being rejected.

Participants responses to the first open- question about international elective were summarized in the following categories:

### Important advice that was repeated frequently

General advice was given repeatedly about different topics including being organized, plan it early on, be initiative, go out of your comfort zone*.*

“Take the risk and go aboard, go out of your comfort zone. It is a very rich experience, help you in improving yourself and discover the world in different way, introduce you to other culture and make you more self-dependent and increase yourself appreciation. The national electives are not handled seriously by students nor by their supervisors.”

“Go with target in mind and put ‘to-do-list’, set objectives beforehand. Be commitment and leave a prominent impact. Try to make connection as it will help when applying for residency program.”

“Start searching for spot as early as possible (plan this at least 2 years ahead, I planned it late and missed an opportunity)”

“Don’t be afraid of asking questions and participation in diverted activities. Be initiative, introduce yourself with confident. Don’t ever underestimate your kills, your knowledge and your abilities. And most importantly, do not get frustrated it if things was not up to your expectations, things will improve with time.”

“Try to get Hands-on experience, it give you a superior ability to learn. But even if you couldn’t and you get observership position try to show interest and introduce yourself with most professionalism and confidence and do not afraid of asking for examining patients and involving in other activities.”

“At the end of the elective, ask for letter of recommendation. It makes a significant improvement in your CV, and get to build connections. Improve your research skills.”

### Selection of location and specialty

It depends on different perspectives, including the country where the student is expecting to apply for training program in and the language of the country.

It is good to read about the country you are planning to visit and have idea about pre-requirements such as visa and language test.

***“*** *Try to choose elective in country you are planning to have your training in future and in specialty you prefer. In addition, try to get into well-known decent institution and raise your standers and goals.”*

“Try not to spend the whole duration of elective in the same department or institution.”

“Read as much as possible about the place or country you're going to in order to save money and time.”

“It is very important to speak and understand the language of the people there, since it is crucial instrument in communicating with medical staff and patients as well, several students had bad experience due to language barrier.”

### On an individual level

Having basic of how to deal with daily life chores like preparing food, washing clothes.

“Learn to wash your clothes before you travel abroad”

“Universities should give students who go abroad for electives an extra week off because of jet lag.”

*“Look for financial support for your trip and/or Visa b*y applying for CAFD travel support”

### Negative reviews about doing elective aboard

Language barrier and cost were the main concerns.

“Don’t do elective abroad as it is not beneficial as it seems and very costy. Students use it for tourism and entertainment and getting to know new people. And the language was a barrier in contacting with patients as well.”

“In Germany, people were completely uncooperative speaking English with the participant.”

In response to the second question, "Do you have any other points you would like to add?" many respondents wondered why they had to pay the university fees for 8 credit hours when they were not taught at the university's hospital. Moreover, another point was emphasized by Hashemite’s graduates regarding restrictions on the selection of the 2 months in which all students start their elective course that resulted in a significant obstacle in obtaining appropriate chances. Others argue that university administration should contribute financially to student support and provide more opportunities and affiliations.

“I wish the results of this study will play a role in changing the stony old rules in Hashemite University which restrict the elective course on June and July.”

“Suggestion to include other purposes for traveling, including CS exam or practicing a language.”

***“*** *University should offer more options to its students in the means of support.”*

“Elective placement outside Jordan is really costy, many of our colleagues deserved that opportunity but couldn't afford it, so our faculties should do something to help them.”

## Discussion

In this study, we evaluated the general value of the elective using 6 items, and significant correlations between these items and the general value of the elective course were identified.

To align their curriculum vitae, students are advised to choose elective specialty choices in the same area as their preferred specialty for their planned postgraduate training program. Van Den Broad et al., 2017 showed that the most popular career interest among medical students is internal medicine and its subspecialties (*n* = 33; 21.6%), followed by family medicine (*n* = 30; 19.6%) and surgery (*n *= 24; 15.7%). In our study, the most popular selected elective courses were general internal medicine (14.8%), followed by general surgery (11.2%), cardiology (35, 9.1%), and pediatrics (6.8%). Many students choose a clinical elective in surgery or internal medicine because they cover many subspecialties, and training in these fields can benefit other careers/specialties as well [13].

Among our participants, 62.6% had a clerkship/hands-on experience in comparison to 36.9% with observership experience. 78% of clerkship/hands on experiences resulted in an evaluation of 4–5 for the general value of the elective, and 5 was the highest average rank (37.6%). On the other hand, 54.2% of observership participants evaluated the general value of an elective between 4 and 5, and 4 was the most common rank selected (37.6%) (Table 5). As clerkships help students participate actively with physicians and patients in the specialty of their interest, it then benefits specialty choice exploration and increases professionalism in practicing medicine and increasing self-efficacy [14]. Surprisingly, direct application to the concerned institution was the primary method of getting placement for 142 (49.9%) students, followed by personal connections (27.5%), which indicates that students get the chance for an elective by applying by to programs more than by relying on friends or relatives for their applications.

A total of 117 (30.4%) participants participated and were engaged in a national elective, of which 43 (11.2%) selected the Ministry of Health Hospital for their elective courses. This can be attributed to the distribution of the Ministry of Health-affiliated hospitals in all provinces of Jordan. In comparison, 268 (69.6%) of the participants had international electives, of which 134 (34.4%) were in the USA. Almost all of the responses to the open-ended question asking for advice on selecting an elective strongly encouraged pursuing an international elective rather than a national one. The reasons behind this are not limited to medical experience but are also related to cross-cultural acceptance from a broader perspective. For example, Queen's University in Canada concluded that international electives benefit medical students' professional and personal development [7]. International experience helps with self-development and maturation, prepares them for upcoming ethical and practical difficulties, informs them about social and cultural determinants of health in the context of global health, and offers valuable experience [15]. Some consider it a "must" to travel internationally. In contrast, others emphasize breaking out of one's comfort zone and venturing out to discover the world and learn medicine in a new way that is culturally competent and sensitive. Exposure to global health issues is also needed among today's trained physicians, which will help in understanding existing and newly emerging global diseases [16].

The USA ranked first among the most favored countries where students prefer to spend their electives. This can be attributed to professional opportunities, an attractive training environment, or political stability [17]. In fact, students participating in observerships, externships or research activities in the USA have the advantage of undertaking the USMLE step 2 clinical skill exam and increasing their competitiveness when applying for residency programs in the future. However, in response to the COVID-19 pandemic, the USMLE step 2 clinical skill exam was stopped [18, 19]. During COVID-19, many hospitals stopped receiving international students, and many students of Jordanian medical schools had their elective cancelled. Some students in class 2020 who had chosen to do the elective during the summer semester or during the first semester were able to do an international elective, but during the 2^nd^ semester, many international elective placements were cancelled, in addition to travel restrictions implemented in Jordan since March 2020.

In response to the open-ended questions, participants showed a high level of awareness, responsibility and maturity. Almost all encouraged undergraduate students to invest their time during their elective efficiently and not to consider it as only a vacation. Many of the responders to the second open-ended question admit that the financial burden is a major barrier for many students to enroll in an international elective. In fact, the USA and the UK are considered much more expensive than Jordan, in which the annual average per capita is $4,028.96 in Jordan compared to $41,535 in the USA in 2020 [20, 21]. Students usually try to manage this issue by choosing less expensive cities and less expensive hospitals for their electives. Some of them go for observerships rather than clerkships. Others, get funds from their families or relatives. Regarding accommodation, many students rent a room in a house through the Airbnb website for their stay or try to share a room with their friends in campus accommodations.

Participants recommended that their schools adopt more flexible policies regarding the timing of elective enrollment and sign agreements with different international medical schools and centers, allowing them to secure elective positions in the future.

### Limitations

The study utilized an online questionnaire, so we could not guarantee that we only received one response per participant, and the questionnaire was mainly distributed among Jordanian graduates currently working in Jordan. Students usually tend to do electives in more than one place, and each carries a different experience and value. Therefore, in this study, we measured the impact of one of the participants’ experiences. The results were for both international and national electives, and no clear comparison between both was performed. More data on cultural and professional development after the elective should be collected. Moreover, the qualitative study was based on open-ended questions instead of interviewing the participants.

In March 2020, quarantine and travel restrictions were applied in Jordan. This might affect the results for the class of 2020 on our questionnaire.

## Conclusion

The elective course provided medical graduates with an enriching environment and cultural experience. The international elective is highly preferred by medical students, especially those who intend to complete their residency abroad. Medical schools are required to adopt more flexible policies regarding the timing of elective engagement and build a network of international affiliations to enhance students’ opportunities to secure elective positions.

## Data Availability

The data that support the findings of this study are available on request from the corresponding author RAA. The data are not publicly available because they could compromise research participant consent.

## Abbreviations

IFMSA: International Federation of Medical Students' Associations
IRB: The Institutional Review Board
N: Frequency
S.D.: Standard Deviation
UK: United Kingdom
USA: United States of America
USMLE: The United States Medical Licensing Examination
